# Noteworthy Factors to Decide Therapeutic Strategy for Carcinoma ex Pleomorphic Adenoma of Parotid Gland: A Preliminary Study Statistical Analysis of 22 Cases from Single Institution

**DOI:** 10.3390/life12111685

**Published:** 2022-10-24

**Authors:** Kosei Mori, Kazuki Yamasaki, Yuki Morimoto, Takashi Kinoshita, Shunichi Asai, Tomoyuki Arai, Tomohisa Iinuma, Syuji Yonekura, Toyoyuki Hanazawa

**Affiliations:** 1 Department of Otorhinolaryngology/Head and Neck Surgery, Chiba University Graduate School of Medicine, Chiba 260-8670, Japan; 2 Department of Head and Neck Surgery, Chiba Cancer Center, Chiba 260-8717, Japan

**Keywords:** carcinoma ex pleomorphic adenoma, CXPA, malignant salivary gland tumor, facial nerve, head and neck cancer

## Abstract

Carcinoma ex pleomorphic adenoma (CXPA) is a rare malignant salivary gland tumor, and its prognosis is determined by the histological progression beyond the adenoma capsule. However, a preoperative evaluation of the histological progression remains challenging, and there is no consensus regarding treatment strategies for CXPA. Herein, we aimed to predict the histological progression preoperatively and develop an appropriate treatment strategy for CXPA. We retrospectively reviewed 22 patients with parotid gland CXPA recorded at our hospital. The clinicopathological characteristics were assessed, and survival analysis was performed. T3≤ or N+ were common in widely invasive CXPA (WICXPA) (*p* < 0.05). A tumor diameter > 40 mm and the N+ status were associated with poor prognosis considering overall survival (OS) and locoregional recurrence rate (LRC) (*p* < 0.05). Patients with facial nerve paralysis exhibited better OS and LRC than those without facial nerve paralysis. More than 90% of patients with WICXPA experienced distant metastases. Meanwhile, there were no cases of recurrence or death due to intracapsular and minimally invasive CXPA. A preoperative advanced T stage or N+ status was suspected as WICXPA. Tumors > 40 mm in size and N+ status necessitate high-intensity local treatment. Facial nerve invasion can be controlled by nerve resection. Postoperative systemic therapy could control distant metastases.

## 1. Introduction

Carcinoma ex pleomorphic adenoma (CXPA) is characterized by the malignant transformation of component cells of pleomorphic adenoma (PA), a benign salivary gland tumor [1]. Salivary gland malignant tumors are rare, accounting for less than 5% of head and neck cancers. CXPA is even rarer, accounting for approximately 10% of salivary gland malignant tumors [1,2]. Reportedly, the frequency of Pas’ malignant transformation ranged between 1.6 and 9.6%, and long disease duration and large tumor size are considered risk factors [3,4,5]. Notably, the parotid gland is the most common site of CXPA, followed by the submandibular gland and palate [6]. Approximately 80% of the malignant component of CXPA is derived from epithelial cells, such as salivary duct carcinoma (SDC) and adenocarcinoma not otherwise specified (NOS) [7,8]. Histologically, CXPA exhibits a stepwise pattern of development, extending from intra-duct to extra-duct, from intra-capsule to extra-capsule, PA, and from intra-salivary gland to extra-salivary gland [9]. Intracapsular CXPA (ICCXPA) and minimally invasive CXPA (MICXPA), with extracapsular invasion within 1.5 mm, reportedly afford a good prognosis, regardless of the histological type of the malignant component, and are classified as low-grade malignancies. In contrast, widely invasive CXPA (WICXPA), with extracapsular invasion ˃ 1.5 mm, exhibits a poor prognosis with frequent recurrence and distant metastasis, classified as high-grade [1,9,10].

Accordingly, the prognosis of CXPA varies greatly, depending on the degree of histological progression. Ideally, CXPA therapy should be tailored according to the level of histological progression; however, it can be challenging to accurately assess histological progression prior to tumor resection in clinical practice. The preoperative diagnosis of parotid gland carcinoma is typically assessed using imaging techniques, such as magnetic resonance imaging, fine needle aspiration, and core needle biopsy; however, these techniques are inadequate for evaluating the capsule/carcinoma location and the precise distance of extracapsular invasion.

In the present study, we analyzed the clinicopathological differences between ICCXPA /MICXPA and WICXPA and derived prognostic factors of CXPA. In addition, we examined the optimization of treatment strategies for CXPA based on observed clinicopathological differences.

## 2. Patients and Methods

We identified 22 patients with parotid gland CXPA recorded at Chiba University Hospital during an 18-year period, from April 2003 to June 2021. We evaluated symptoms at the initial examination by examining past medical records. The patients’ age ranged between 38 and 87 years (median, 60 years), and the observation periods ranged from 6 to 178 months (median, 62.5 months).

All patients were staged according to the 2017 Union for International Cancer Control classification, and histological classification was evaluated according to the 2017 World Health Organization classification.

The treatment strategy for parotid gland carcinoma at our hospital was as follows: the primary tumor was resected by total parotidectomy, regardless of tumor grade. The facial nerve was resected in the following cases: clinically, T3≤, N+, or preoperative pathology suspected of being high-grade. However, if preoperative facial nerve paralysis was absent and the intraoperative findings showed a sufficient margin between the facial nerve and tumor, the facial nerve was preserved. Comprehensive lateral neck dissection was performed in clinical N+ cases, and elective neck dissection at levels I, II, III, and VA was performed even in N0 cases. Postoperative radiation therapy was administered in the following cases: high-grade, positive cervical lymph nodes, positive resection margins, and positive nerve/vascular/lymphatic invasion.

For immunohistochemical staining, we evaluated samples from all patients as described in previous reports. Human epidermal growth factor receptor 2 (HER2) was scored from 0 to 3+ according to the criteria of the American Society of Clinical Oncology/College of American Pathologists Clinical Practice Guideline, with 2+ or 3+ classified as positive [11]. Androgen receptor (AR) was classified as positive when AR expression was detected in ˃1% of nuclei [11].

Unpaired *t*-tests were used to compare quantitative variables between the two groups. The chi-squared and Fisher’s exact tests were employed to examine the association between categorical variables. The Kaplan-Meier method was used to calculate survival rates, and the log-rank test was used for testing significance. All statistical analyses were performed using EZR version 1.55. Statistical significance was set at *p* < 0.05.

This study was approved by the Ethical Review Committee of Chiba University Hospital (M10133).

## 3. Results

### 3.1. Clinical Characteristics and TNM Classification

We identified 22 patients with parotid gland CXPA, including 5 with ICCXPA, 2 with MICXPA, and 15 with WICXPA. We compared the clinical characteristics and pathologic TNM classification, considering the first two types as the same group, namely “ICCXPA/MICXPA”.

There were no significant differences in the mean age and sex ratio between the two groups. At the initial examination, pain, mobility failure, and facial nerve paralysis were noted in 47, 42, and 40% of patients with WICXPA and 29, 17, and 0% of patients with ICCXPA/MICXPA, respectively. Patients with ICCXPA/MICXPA tended to exhibit fewer symptoms than those with WICXPA, with no facial nerve paralysis, and there was no significant difference between the two groups considering symptoms at the initial examination. The mean maximum tumor diameter was 46.2 and 33.3 mm in patients with WICXPA and ICCXPA/MICXPA, respectively. Among patients with WICXPA, eight (53%) had a tumor diameter of >40 mm, and seven (47%) presented a diameter of ≤ 40 mm. Six of the seven patients with a maximum tumor diameter of ≤ 40 mm exhibited facial nerve paralysis at the initial examination.

Fourteen (93%) and two (29%) patients presented advanced T stage (T3 or T4) in the WICXPA and ICCXPA/MICXPA groups, respectively. The difference between the two groups was statistically significant (*p* < 0.05).

Cervical lymph node metastasis was observed in 11 (73%) patients with WICXPA. Conversely, no patient with ICCXPA/MICXPA exhibited cervical lymph node metastasis, and the difference between the two groups was significant (*p* < 0.05) (Table 1).

### 3.2. Pathologic Characteristics

We examined the CXPA of the parotid gland histopathologically using surgically resected samples. The most common histological type of the malignant component of CXPA was SDC in 14 patients (63%), followed by adenocarcinoma NOS in 2 patients (9%), and myoepithelial carcinoma in 2 patients (9%). There were no differences in the histology of malignant components between the ICCXPA/MICXPA and WICXPA groups (Table 2).

Nerve, vascular, and lymphatic invasions were observed in 76, 64, and 43% of patients with WICXPA, respectively. Conversely, patients with ICCXPA/MICXPA showed no nerve, vascular, or lymphatic invasion. There were significant differences in nerve and vascular invasion between the two groups (*p* < 0.05). In addition, we noted no differences in positivity rates of HER2 and AR between the ICCXPA/MICXPA and WICXPA groups. The HER2 and AR positivity rates for all CXPA were 50 and 85%, respectively (Table 3).

### 3.3. Treatment

The primary lesion was treated by performing total parotidectomy in 7 patients and extended parotidectomy in 14 patients, with a complete resection of the parotid glands performed in almost all patients. One patient underwent partial parotidectomy as a less invasive technique, given that the patient was ˃ 80 years of age. Elective neck dissection was performed in 10 patients, and comprehensive neck dissection was performed in 9 patients. All three patients without neck dissection were classified as low-grade and did not receive adjuvant therapy; to date, none of these three patients experienced recurrence. Of the 22 patients, 13 underwent complete resection of the facial nerve, and 1 patient underwent partial facial nerve resection. Immediate reconstruction using the sural nerves or greater auricular nerves was performed in all 14 patients who underwent facial nerve resection. Twelve patients with WICXPA underwent postoperative radiation therapy. The mean radiation dose was 62.5 Gy. Six patients received chemoradiotherapy with a combination of cisplatin and 5-fluorouracil, cisplatin, carboplatin, and S-1. Three patients who did not receive radiation therapy received chemotherapy with S-1 only (Table 3).

### 3.4. Follow-up and Survival

**Figure 1:**
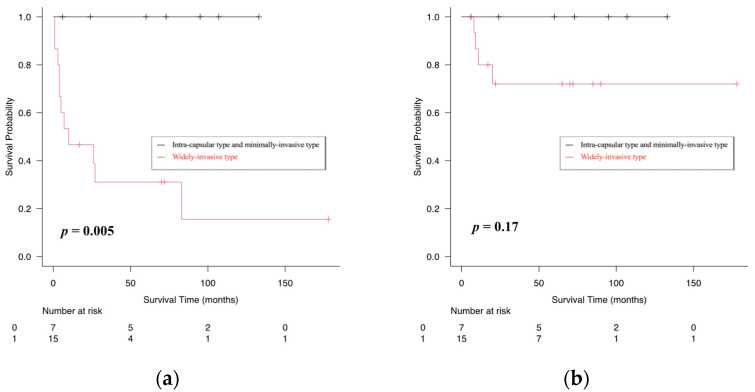
Kaplan–Meier curves showing comparison of disease-free (**a**), overall (**b**) survival rates between intra-capsular/minimally invasive type and widely invasive type of carcinoma ex pleomorphic adenoma (CXPA).

The follow-up period ranged from 6 to 178 months (median, 62.5 months). Four patients (18%) with WICXPA died during the follow-up period. All four deaths in the WICXPA group experienced local or regional recurrence, potentially related to the primary disease. The five-year disease-free survival (DFS) and overall survival (OS) rates were 100 and 31.1% (*p* < 0.05) for ICCXPA/MICXPA and 100 and 72% for WICXPA, respectively. No recurrence or death was documented in patients with ICCXPA/MICXPA (Figure 1).

**Figure 2:**
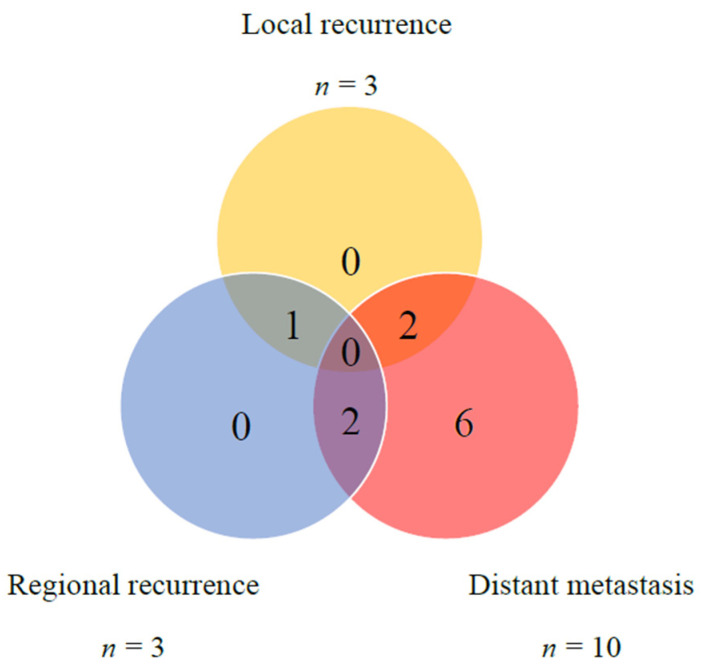
Distribution of local, regional, and distant failures in 22 CXPA cases.

Postoperative recurrence was documented in 11 (50%) patients during the follow-up period. Distant metastases were the most frequently observed, noted in 10 patients. The median time to distant metastasis was 6 months. Local or regional recurrence was observed in five patients. One patient exhibited both local and regional recurrence, two experienced only local recurrence, and two experienced only regional recurrence. The median time to locoregional recurrence was 3 months (Figure 2).

### 3.5. Kaplan-Meier Analysis of Prognostic Factors

A Kaplan–Meier analysis of all 22 patients with CXPA revealed significant differences in DFS considering sex, facial nerve paralysis, N stage (N0 vs. N+), and pathological findings of nerve and vascular invasion (*p* < 0.05). However, we noted no significant differences in DFS for age (≤60 vs. >60 years), T stage (≤T2 vs. ≤T3), and tumor diameter (≤40 mm vs. >40 mm).

Next, we examined prognostic factors impacting OS and detected significant differences in age, tumor diameter, and N stage (*p* < 0.05). However, no significant differences in OS were found for sex, facial nerve paralysis, T stage, and pathological findings of nerve invasion and/or vascular invasion (Table 4).

In addition, we compared locoregional control (LRC) rates and detected significant differences between the two groups considering age (*p* < 0.05), tumor diameter (*p* < 0.01), N stage (*p* < 0.01), and vascular invasion (*p* < 0.05). However, no significant differences were detected considering sex, facial nerve paralysis, T stage, and nerve invasion (Table 5).

## 4. Discussion

Herein, we aimed to analyze the clinicopathological characteristics and prognostic factors of CXPA, for which a preoperative evaluation of histological progression can be challenging. Furthermore, we attempted to predict the precise grade and establish an appropriate treatment according to the grade. Previous reports on CXPA using population-based databases have been limited, given the difficulties in assessing uncoded data [12,13]. The present study is a case report investigation from a single institution with consistent treatment and follow-up, with targeted primary lesions confined to the parotid gland among salivary glands; hence, there were few differences in the anatomical extension pattern. Therefore, this report may be useful in establishing treatment strategies for CXPA.

In order to explore the differences in clinical findings between ICCXPA/MICXPA and WICXPA, we compared patient data, maximum tumor diameter, and the triad of malignancy, pain, mobility failure, and facial nerve paralysis, none of which significantly differed in the present study. Although a long PA duration is considered a risk factor for malignant transformation [4], there was no difference in age at diagnosis between each malignancy grade. This suggests that age may not be a predictor of malignancy grade. Among the triad of malignancies, facial nerve paralysis was present in approximately 40% of patients with WICXPA but not in those with ICCXPA/MICXPA. We did not detect any significant differences in facial nerve paralysis between the two groups; however, preoperative facial nerve paralysis might reflect the degree of tissue extension; therefore, it could be useful in predicting malignancy grade.

Regarding TNM classification, the ICCXPA/MICXPA group comprised more patients with early T (≤T2) and N0. In contrast, patients with WICXPA were diagnosed with more advanced T (T3≤) and N+ stages, and the difference between the two groups was significant. These results suggest that T3≤ and N+ indicate advanced histological progression and may be useful in predicting malignancy grades.

Cervical lymph node metastases were observed in 70% of patients with WICXPA. In contrast, all patients with ICCXPA/MICXPA showed N0 disease. In patients with preoperative N+, high-intensity treatment should be implemented for suspected WICXPA. Extended resection, including facial nerve resection, comprehensive lateral neck dissection, and postoperative radiation therapy, should be recommended as potential treatment strategies for high-grade parotid carcinoma [14,15,16].

Considering T classification, patients with WICXPA were diagnosed as T3 or more advanced stages, with approximately 50% of these patients presenting a maximum tumor diameter >40 mm; the remaining patients were diagnosed as T4 due to facial nerve invasion. A tumor diameter exceeding 40 mm is considered to be the determining factor for T3, and the presence of facial nerve paralysis indicates stage T4a, according to the TNM classification of the major salivary gland. The conventional TNM classification was considered to efficiently reflect the histological progression of CXPA.

In the present study, almost all the patients underwent total parotidectomy and neck dissection. Considering WICXPA, the facial nerve was resected, and postoperative adjuvant therapy, including radiation therapy, was performed. Therefore, patients with WICXPA at our hospital were treated intensely for local lesions using the current standard therapy. However, local and regional recurrences were detected in five patients, and four of these patients died during the observation period. Given that the only deaths in the present study were these four patients, it can be suggested that locoregional recurrence significantly impacted survival.

Considering prognostic factors that impact patient survival in CXPA, a maximum tumor diameter >40 mm and N+ showed poor survival with significant differences in both OS and LRC. Increased locoregional recurrence was attributed to difficulties in controlling extensive micrometastases arising from large primary tumors (>40 mm) or preoperatively detected cervical lymph node metastasis. In contrast, we noted no significant differences in OS and LRC for T3≤, facial nerve paralysis, or nerve invasion. Regarding facial nerve paralysis, positive cases showed better OS and LRC than negative cases. This result suggests that facial nerve resection could control local recurrence even in advanced T stage >T3 with facial nerve invasion. A previous population-based study reported that T3≤ was an independent prognostic factor for OS in 126 patients with parotid gland CXPA [17]. Conversely, our study suggested that an advanced T stage (T3≤) did not typically indicate a poor prognosis and that an appropriate margin resection might control local recurrence and improve the prognosis for facial nerve paralysis. Interestingly, our study suggests that facial nerve resection may improve the prognosis of patients confirmed with T4a stage.

Regarding ICCXPA/MICXPA, patients did not experience recurrent metastasis. In the present study, we detected no patients with occult lymph node metastasis owing to the prophylactic neck dissection for ICCXPA/MICXPA. In addition, some patients with ICCXPA/MICXPA did not undergo neck dissection, and none experienced recurrence or metastases. Typically, the prognosis for ICCXPA/MICXPA is good, and the complete resection of local lesions results in almost no recurrence or metastasis [9]. For ICCXPA/MICXPA, a de-escalation of the treatment, such as the omission of neck dissection, could be considered.

In summary, patients with CXPA presenting T3≤ or N+ were suspected to be WICXPA. Tumor diameter and cervical lymph node metastasis were considered important adverse prognostic factors, and a tumor diameter >40 mm or N+ may imply the need for high-intensity local treatment with sufficient margins to avoid locoregional recurrence. Furthermore, facial nerve resection could grant local control and improve prognosis in patients preoperatively diagnosed with T4 and facial nerve paralysis, which were suspected as WICXPA. Since this study reviewed one histological subtype included in a rare malignancy, the number of cases was limited. Therefore, we focused only on the prediction of histological progression of CXPA, which is directly related to the treatment strategy and local treatment, which has provided evidence of the treatment for parotid gland malignant tumors. Several points could not be examined in this study, such as risk factors for malignant transformation of PA, preoperative pathological diagnosis, and the prognostic influence of histological type of malignant component, but these are expected to be further investigated by accumulating more cases in the future. In addition, our continued follow-up is needed to examine the longer-term prognosis, especially in cases of distant metastasis. In this study, only univariate analysis of prognostic factors could be performed. Although we considered that multivariate analysis was not statistically valid due to the small number of cases, it is necessary to conduct multicenter studies to accumulate more cases in the future. This study was retrospective, and it is desirable to adjust for confounding factors using multivariate analysis and to eventually confirm the results with multicenter prospective studies.

Recurrences or metastases were detected in 11 of the 15 patients with WICXPA, of which more than 90% were distant metastases. SDC is a salivary gland malignancy with distant metastasis and a poor prognosis [18]. SDC was the most common histology of the malignant component of CXPA, accounting for more than 60% of the patients in the present study. Considering WICXPA, it has been suggested that the malignant component may invade beyond the capsule, and the clinical course might be equivalent to that of de novo carcinoma of the malignant component. Accordingly, WICXPA may present more distant metastases and a poorer prognosis. Histopathologically, SDC shows invasive potential, with frequent nerve and lymphatic invasion [19]. Consistent with the findings of the present study, WICXPA has been associated with strong invasive potential. Additionally, the HER2-positive rate of SDC was found to be approximately 40%, and the AR-positive rate was reported to be > 90% [20,21]. Herein, the HER2-positive rate of CXPA was 50%, and that of AR was 85%, similar to that observed in SDC. These results suggest that SDC and WICXPA may exhibit markedly similar pathogenesis. The efficacy of HER2-targeted therapy and androgen deprivation therapy for recurrent/metastatic or unresectable salivary gland cancer has been recently reported [11,21,22]. Currently, the efficacy of postoperative chemotherapy for parotid carcinoma has not been established; however, these new treatments might effectively control postoperative distant metastases of CXPA. Developing new postoperative systemic therapies to control distant metastases is urgently required.

## 5. Conclusions

Herein, we analyzed 22 patients with parotid gland CXPA. CXPA presented distinct prognoses depending on the degree of histological progression. We detected no recurrence or deaths in patients with ICCXPA or MICXPA, whereas almost all patients with WICXPA experienced recurrence or metastasis, indicating a poor prognosis. TNM classification efficiently reflected the histological progression or malignancy grade for CXPA. Preoperative advanced T stage or N+ suggested a high-grade malignancy, and WICXPA was suspected in these patients. A tumor diameter >40 mm and N+ status were poor prognostic factors for OS and LRC. Facial nerve resection could effectively control local recurrence in patients with facial nerve paralysis. In order to improve prognosis, postoperative systemic therapy for CXPA, especially WICXPA, is expected to control distant metastasis.

## Figures and Tables

**Table 1:** Preoperative findings and pathological TNM stages of parotid gland CXPA.

	Intra-Capsular Type (*n* = 5) and Minimally Invasive Type (*n* = 2)		Widely Invasive Type (*n* = 15)		
Features	No. of Patients	Percentage	No. of Patients	Percentage	*p* Value
Age [year, average]	[59.4]		[62.9]		0.58
≤60	4	57.1	7	46.7	1.0
>60	3	42.9	8	53.3	
Sex					
Female	3	42.9	5	33.3	1.0
Male	4	57.1	10	66.7	
Pain					
Yes	2	28.6	7	46.7	1.0
No	5	71.4	8	53.3	
Mobility failure					
Yes	1	14.3	5	33.3	1.0
No	5	71.4	7	46.7	
Unknown	1	14.3	3	20.0	
Facial nerve paralysis					0.36
Yes	0	0.0	6	40.0	
No	7	100.0	9	60.0	
Tumor diameter [mm, average]	[33.3]		[46.2]		0.16
≤40 mm	5	71.4	7	46.7	0.38
>40 mm	2	28.6	8	53.3	
T stage					
T1/T2	1/4	71.4	0/1	6.7	0.013
T3/T4	2/0	28.6	6/8	93.3	
N stage					
N0	7	100.0	4	26.7	0.012
N1/N2/N3	0/0/0	0.0	2/8/1	73.3	

**Table 2:** Histologic subtypes of malignant components of parotid gland CXPA.

	Histologic Subtype	No. of Patients
Intra-capsular type and Minimally invasive type	Salivary duct carcinoma	5
	Myoepithelial carcinoma	1
	Squamous cell carcinoma	1
Widely invasive type	Salivary duct carcinoma	9
	Adenocarcinoma NOS	2
	Myoepithelial carcinoma	1
	Mucoepidermoid carcinoma	1
	Adenoid cystic carcinoma	1
	Squamous cell carcinoma	1

**Table 3:** Operative and postoperative findings of parotid gland CXPA.

	Intra-Capsular Type (*n* = 5) and Minimally Invasive Type (*n* = 2)		Widely Invasive Type (*n* = 15)		
Features	No. of Patients	Percentage/ Positivity Rate	No. of Patients	Percentage/ Positivity Rate	*p* Value
Primary tumor surgery					
Extended parotidectomy	2	28.6	12	80.0	
Total parotidectomy	4	57.1	3	20.0	
Partial parotidectomy	1	14.3	0	0.0	
Facial nerve					
Excised	2	28.6	12	80.0	
Preserved	5	71.4	3	20.0	
Neck dissection					
Comprehensive	1	14.3	8	53.3	
Elective	3	42.9	7	46.7	
None	3	42.9	0	0.0	
Postoperative therapy					
Radiation	0	0.0	6	40.0	
Radiation + Chemotherapy	0	0.0	6	40.0	
Chemotherapy	0	0.0	3	20.0	
None	7	100.0	0	0.0	
Surgical margin					
Positive	0	0.0	1	6.7	1.0
Negative	7	100.0	14	93.3	
Neural invasion					
Positive	0	0.0	9	69.2	0.014
Negative	7	100.0	4	30.8	
Unknown	0		2		
Vascular invasion					
Positive	0	0.0	9	64.3	0.021
Negative	7	100.0	5	35.7	
Unknown	0		1		
Lymphatic invasion					
Positive	0	0.0	6	42.9	0.18
Negative	7	100.0	8	57.1	
Unknown	0		1		
HER2					
Positive	1	50.0	4	50.0	1.0
Negative	1	50.0	4	50.0	
Unknown	5		7		
AR					
Positive	3	100.0	8	80.0	1.0
Negative	0	0.0	2	20.0	
Unknown	4		5		

**Table 4:** Univariate analysis of factors influencing 5-year disease-free survival/5-year overall survival of parotid gland CXPA.

		No. of Patients	5Y-DFS	*p* Value	5Y-OS	*p* Value
Age	≤60	11	59.7	0.22	100.0	0.045
	>60	11	43.6		63.6	
Sex	Female	8	87.5	0.02	87.5	0.59
	Male	14	28.9		76.2	
Facial nerve paralysis	No	16	67.7	0.02	72.7	0.19
	Yes	6	16.7		100.0	
Tumor diameter	≤40 mm	12	55.6	0.61	100.0	0.02
	>40 mm	10	50.0		58.3	
T stage	≤T2	6	83.3	0.10	100.0	0.23
	T3≤	16	40.2		73.9	
N stage	N0	11	90	<0.001	100.0	0.04
	N+	11	18.2		62.3	
Neural invasion	Negative	9	80.8	0.006	78.7	0.61
	Positive	11	22.2		88.9	
Vascular invasion	Negative	9	79.5	<0.001	90.0	0.15
	Positive	12	11.1		66.7	

**Table 5:** Univariate analysis of factors influencing 5-year locoregional control rates.

		No. of Patients	5Y-LRC	*p* Value
Age	≤60	11	100.0	0.018
	>60	11	54.5	
Sex	Female	8	87.5	0.35
	Male	14	67.5	
Facial nerve paralysis	No	16	74.5	0.77
	Yes	6	75.0	
Tumor diameter	≤40 mm	12	100.0	0.008
	<40 mm	10	50.0	
T stage	≤T2	6	100.0	0.16
	T3≤	16	67.7	
N stage	N0	11	100	0.008
	N+	11	48.5	
Neural invasion	Negative	9	80.8	0.69
	Positive	11	74.1	
Vascular invasion	Negative	9	90.9	0.02
	Positive	12	50.0	

## Data Availability

The data presented in this study are available upon request from the corresponding author. The data are not publicly available due to privacy.
